# Antibiotic agrochemical treatment reduces endosymbiont infections and alters population dynamics in leafminers, thrips, and parasitoid wasps

**DOI:** 10.3389/fmicb.2025.1605308

**Published:** 2025-06-10

**Authors:** Yuta Ohata, Yohsuke Tagami

**Affiliations:** ^1^United Graduate School of Agricultural Science, Gifu University, Gifu, Japan; ^2^Graduate School of Agriculture, Shizuoka University, Shizuoka, Japan

**Keywords:** endosymbiont, antibiotic agrochemical, pest control, *Wolbachia*, natural enemy insect

## Abstract

**Introduction:**

The agricultural pests *Liriomyza trifolii* (Diptera: Agromyzidae) and *Hercinothrips femoralis* (Thysanoptera: Thripidae) harbor the endosymbiont *Wolbachia*, which induces cytoplasmic incompatibility and thelytokous parthenogenesis (asexual reproduction of female offspring without fertilization), respectively. The parasitoid *Neochrysocharis formosa* (Hymenoptera: Eulophidae), a natural enemy of leaf miners, is infected with *Rickettsia*, which also induces thelytokous parthenogenesis. Although symbionts can be eliminated in laboratory settings using antibiotics mixed with physical manipulation, the effects of agrochemical antibiotics designed for plant disease control on these insects and their symbionts remain unexplored. This study investigated the effects of MycoShield, a commercially available agrochemical containing 17% oxytetracycline, on symbiont-infected populations of these three insect species.

**Methods:**

MycoShield was applied to kidney bean plants or mixed into honey to expose *L. trifolii*, *H. femoralis*, and *N. formosa* to oxytetracycline. Offspring were screened for symbiont presence using PCR, and infection frequencies were compared across treatment concentrations. Additionally, *H. femoralis* populations were monitored in caged conditions under continuous exposure to treated plants.

**Results:**

At standard concentrations (1,000-fold dilution), MycoShield eliminated *Wolbachia* from *L. trifolii* and *H. femoralis*, resulting in *L. trifolii* producing uninfected offspring and *H. femoralis* producing only uninfected males. Similarly, *Rickettsia* was eliminated from *N. formosa* when adults ingested MycoShield-mixed honey. Additionally, *N. formosa* appeared to ingest the antibiotic indirectly by parasitizing *L. trifolii* larvae that had fed on treated leaves. Symbiont elimination was dose-dependent. Long-term exposure led to a substantial reduction in *H. femoralis* populations. Two out of eleven cages experienced complete extinction by day 100, likely due to genetic drift resulting from severe reproductive bottlenecks.

**Discussion:**

These findings demonstrate the potential of agrochemical antibiotics such as MycoShield as insecticidal agents targeting symbiont-mediated reproduction, with possible applications in sterile insect techniques. Further research is required to optimize efficacy and assess feasibility under field conditions.

## 1 Introduction

The leaf miner *Liriomyza trifolii* (Burgess) (Diptera: Agromyzidae) is a globally distributed pest that infests various host plants, including vegetables and ornamental flowers (Seal et al., 2002; Masetti et al., 2004). The parasitoid *Neochrysocharis formosa* (Westwood) (Hymenoptera: Eulophidae), a natural enemy of leaf miners, is native to regions where these pests are prevalent (Arakaki and Kinjo, 1998; Konishi, 1998). *N. formosa* parasitizes the larvae of *L. trifolii*, ultimately killing the host. This parasitic behavior enables *N. formosa* to serve as an effective biological control agent and, in Japan, it is now commercially available for managing *Liriomyza* species. Another significant pest, *Hercinothrips femoralis* Reuter (Thysanoptera: Thripidae), commonly known as banded greenhouse thrips, is a polyphagous insect with a cosmopolitan distribution (Houston et al., 1991; Trdan et al., 2007). These three insect species harbor endosymbiotic bacteria such as *Wolbachia* and *Rickettsia* in Japan and other regions (Adachi-Hagimori et al., 2008; Tagami et al., 2006b; Kumm and Moritz, 2008).

*Wolbachia* is a well-studied endosymbiotic bacterium that manipulates host reproduction through various mechanisms, including cytoplasmic incompatibility (Werren, 1997), feminization of genetic males (Bouchon et al., 1998), male killing (Hurst and Jiggins, 2000), and thelytokous parthenogenesis, a form of parthenogenesis in which unfertilized eggs develop into females (Stouthamer et al., 1990; Stouthamer and Kazmer, 1994). In *L. trifolii*, *Wolbachia* induces cytoplasmic incompatibility, a reproductive barrier in which crosses between infected males and uninfected females result in embryo mortality, thereby reducing reproductive success (Tagami et al., 2006b; Kumm and Moritz, 2008). However, this mechanism does not affect the sex ratio of the offspring.

In contrast, in *H. femoralis*, *Wolbachia* induces thelytokous parthenogenesis, a form of parthenogenesis in which unfertilized eggs develop into females, resulting in strongly female-biased populations (Kumm and Moritz, 2008). Similarly, in *N. formosa*, the endosymbiont *Rickettsia* induces thelytokous parthenogenesis, leading to highly female-skewed populations (Hagimori et al., 2006) that contribute to the effectiveness of this species as a biological control agent. Notably, *H. femoralis* females uninfected with *Wolbachia* and *N. formosa* females uninfected with *Rickettsia* produced only male offspring, and these males do not have a function to reproduce, highlighting the essential role of these endosymbionts in reproduction. In pest species like *H. femoralis*, disrupting symbiont-mediated reproduction may aid in population suppression.

In contrast, for beneficial parasitoids like *N. formosa*, which are used as biological control agents against *L. trifolii*, the loss of symbiont-induced thelytoky parthenogenesis could severely reduce female production and compromise control efficacy. Therefore, it is crucial to assess how agricultural treatments, such as antibiotic applications, might differentially affect symbiont-dependent reproduction in both pests and their natural enemies.

The widespread use of agricultural pesticides, including insect growth regulators, biopesticides targeting *Bacillus thuringiensis*, and chemical classes such as organophosphates, carbamates, neonicotinoids, organocoppers, and organic sulfur compounds, has been central to pest control strategies (Siddall, 1976; Whalon and Wingerd, 2003; Souto et al., 2021; Sharma et al., 2019; Zhang, 2018; Araújo et al., 2023). Antibiotics are also used in agriculture to manage plant diseases (Stockwell and Duffy, 2012). One such bactericide, MycoShield (Maruwa Biochemical, Tokyo, Japan), contains 17.0% oxytetracycline, a tetracycline derivative used to control bacterial diseases such as fire blight (*Erwinia amylovora*) and infections caused by *Pseudomonas* and *Xanthomonas* species (Copping and Duke, 2007). This antibiotic is also effective against diseases caused by mycoplasma-like organisms (Ishiie et al., 1967) and is applied to stone and pome fruits, as well as turfgrass (Copping and Menn, 2000).

Despite the prevalence of bacterial endosymbionts in many insect species, the effects of antibiotic bactericides on symbionts and their hosts are unclear. Therefore, this study aimed to investigate the effects of MycoShield on *L. trifolii*, *H. femoralis*, and *N. formosa*, with a specific focus on (i) the direct effects of MycoShield on these insects, (ii) the uptake routes of MycoShield in *L. trifolii* and *N. formosa*, (iii) the relationship between MycoShield concentration and its effect on infected insects, and (iv) the potential use of MycoShield to eradicate *H. femoralis* populations.

## 2 Materials and methods

### 2.1 Insects

*Liriomyza trifolii* and *N. formosa* were collected from kidney bean leaves in Iwata, Shizuoka, Japan, in 1991 and placed in cages (width 30 cm, depth 30 cm, height 40 cm). Each cage contained three kidney bean plants, which served as host plants when *L. trifolii* adults emerged.

Kidney bean leaves in the field were collected for *N. formosa*, and *N. formosa* individuals were selected based on morphological characters when leaf miner parasitoids emerged. *N. formosa* were reared in cages with *L. trifolii* larvae as hosts.

*Hercinothrips femoralis* was collected from a beach lily (*Crinum asiaticum*) in Iwata, Shizuoka, Japan, in 2005 and reared on kidney bean plants in a cage. When host plants were severely damaged, *H. femoralis* populations were transferred to new cages containing new kidney bean plants.

All three insect species were reared under consistent environmental conditions (25°C, 16 h light:8 h dark photoperiod, relative humidity 70 ± 5%, with light provided by MLR-351, SANYO, Osaka, Japan). Morphological identification of each species was performed based on criteria described previously (Konishi, 1998). The initial *Wolbachia* and *Rickettsia* infection status was verified by PCR prior to the experiments.

### 2.2 Detection of *Wolbachia* and *Rickettsia* and distinguishing between sex

Diagnostic PCR detection of bacterial endosymbionts was performed using a thermal cycler (TP600, TAKARA, Japan) as previously described (Tagami et al., 2006b; Hagimori et al., 2006; Tagami et al., 2006a). *Wolbachia* was detected by amplifying the *wsp* gene using the primers wsp81F (5′-TGGTCCAATAAGTGATGAAGAAAC-3′) and wsp691R (5′-AAAAATTAAACGCTACTCCA-3′) (Braig et al., 1998). *Rickettsia* infection was assessed by targeting the bacterial 16S rRNA gene with the primers NforRick1 (5′-AGTGAGTGATGAAGGCCCTA-3′) and NforRick2 (5′-GGAATTCCATCATCCTCTACTAC-3′) (Tagami et al., 2006a). The PCR conditions (reaction components and thermal cycling profiles) followed those established in the cited literature.

Host sex was identified based on adult morphological characteristics, particularly the structure of the sexual organs, in all three species.

### 2.3 Effect of MycoShield on *L. trifolii*, *H. femoralis*, and *N. formosa*

Prior to MycoShield treatment, the infection status of each insect species was confirmed by PCR using 30 representative individuals from the laboratory-reared lines. All *L. trifolii* and *H. femoralis* individuals tested were found to be 100% infected with *Wolbachia*, and all *N. formosa* females were 100% infected with *Rickettsia*.

To assess the effect of short-term antibiotic exposure, adult insects were placed in cages containing MycoShield-treated plants for 24 h. MycoShield was prepared at a standard concentration (1,000-fold dilution with water) and sprayed evenly onto three kidney bean plants, each bearing four leaves. After the 24-h exposure period, three pairs of *L. trifolii* or *H. femoralis* were introduced into separate cages and allowed to reproduce. Once the adults of the next generation emerged, six individuals were randomly collected from each cage and screened for *Wolbachia* infection using PCR. Each experiment was repeated five times.

For *N. formosa*, five cages were prepared, and a droplet of honey mixed with MycoShield (1,000-fold dilution) was provided as food for 24 h. After this exposure period, three kidney bean plants parasitized by *L. trifolii* larvae were introduced into each cage. The *Rickettsia* infection ratio was determined by analyzing 30 adult individuals (six individuals per cage) of *N. formosa* from the next generation using PCR.

### 2.4 MycoShield uptake routes of *L. trifolii* and *N. formosa*

Understanding the uptake routes of MycoShield by *L. trifolii* and *N. formosa* is essential for the application of antibiotic agrochemicals to manage bacteria-infected pest insects and their natural enemies.

#### 2.4.1 MycoShield uptake route of *L. trifolii*

The uptake route of *L. trifolii* was initially analyzed. As *L. trifolii* does not feed directly on the surface of kidney bean leaves, we hypothesized that MycoShield is primarily ingested by larvae feeding on the mesophyll rather than by adults. Two separate experiments were conducted to test this hypothesis.

First, MycoShield was prepared at a standard concentration (diluted 1,000-fold with water) and sprayed onto 12 leaves from three kidney bean plants. Three pairs of *L. trifolii* adults were introduced into cages containing the treated plants. After one and three days, the adults were transferred to a new cage containing three untreated kidney bean plants. Eggs laid on untreated plants were collected, and the infection status of *Wolbachia* in the offspring was analyzed. The experiments were conducted under controlled conditions (25°C, 16 h light:8 h dark photoperiod).

Next, three pairs of *L. trifolii* adults were introduced into a cage containing three untreated kidney bean plants to examine the uptake of MycoShield by the larvae. After 24 h, the plants with *L. trifolii* eggs were collected. These plants were then sprayed with MycoShield at the standard concentration and placed in a new cage under controlled conditions (25°C, 16 h light:8 h dark photoperiod). Larvae were collected at four, six, and eight days after treatment, and their *Wolbachia* infection status was analyzed.

#### 2.4.2 MycoShield uptake route of *N. formosa*

*Neochrysocharis formosa* does not feed on kidney bean leaves; therefore, it possibly ingests MycoShield through *L. trifolii* larvae, which consume MycoShield-treated leaves. Adult *N. formosa* individuals less than 24 h after eclosion were used for these experiments, and all experiments were done at 25°C and a 16 h light:8 h dark photoperiod.

First, it was confirmed that *N. formosa* adults did not directly ingest MycoShield from the leaves. MycoShields were sprayed onto three unparasitized plants (12 leaves in total) inside a cage, and six *N. formosa* adults were released. Then these adults were moved to new cages on days one-, three-, five-, and seven-day days later, which introduced untreated kidney bean parasitized by *L. trifolii*. The infection and male ratios of adult individuals in the next generation were analyzed (Supplementary Figure 1A).

*L. trifolii* larvae immediately die when parasitized by *N. formosa*. Second, *N. formosa* is expected to ingest *L. trifolii* larvae if *L. trifolii* larvae die before being sprayed with MycoShield. MycoShield was sprayed after parasitizing *N. formosa*, and the infection ratio during the pupal stage of *N. formosa* was determined (Supplementary Figure 1B).

Third, whether *N. formosa* could ingest MycoShield through adult host feeding was investigated. MycoShield was sprayed onto kidney bean plants that had been oviposited by *L. trifolii* five days earlier. Three *N. formosa* individuals were introduced into the cages. *N. formosa* adults were transferred to new, non-sprayed plants parasitized by *L. trifolii* on days one, three, five, and seven. Approximately 24 h later, *N. formosa* eggs were collected, and their infection ratio was analyzed. The experiment was repeated six times (Supplementary Figure 1C).

Fourth, the combined routes of MycoShield uptake by *N. formosa* were investigated. Five pairs of *L. trifolii* adults were introduced into the cages containing kidney bean plants. After five days, the oviposited eggs had developed into larvae, and the plants were sprayed with MycoShield before being moved to a new cage. *N. formosa* adults were introduced in this new cage, where they could only ingest MycoShield through host feeding, whereas *N. formosa* larvae consumed *L. trifolii* larvae that had absorbed MycoShield. Emerging *N. formosa* adults were collected, and their infection and male ratios were analyzed (Supplementary Figure 1D).

### 2.5 Effect of MycoShield concentration on *L. trifolii*, *H. femoralis*, and *N. formosa* populations

MycoShield solutions were prepared at different concentrations and diluted with water (100-, 1, 000-, 10, 000-, 100, 000-, 1,000,000-fold) with an untreated control. Two kidney bean plants (eight leaves in total) were used in each experiment. Once the plants had grown sufficiently, the MycoShield solutions were sprayed evenly onto their leaves, and these plants were placed inside a cage.

Three pairs of *L. trifolii* adults were randomly selected and placed in cages. Six *H. femoralis* and *N. formosa* adults were introduced into the cage. For *N. formosa*, two kidney bean plants that had been oviposited with *L. trifolii* were used. Six adult *N. formosa* individuals were introduced into the cage when oviposited *L. trifolii* eggs developed into larvae. The infection and male ratios in the next generation of adults were analyzed.

### 2.6 Effect of the MycoShield on *H. femoralis* population dynamics

Twelve kidney bean plants in four pots were prepared, which were sprayed with a standard concentration of MycoShield (diluted 1,000-fold with water) and placed inside a cage. Twenty adult female *H. femoralis* were introduced into cages. Larvae, adult females, and adult males were counted every 20 days.

After each count, new plants treated with a standard concentration of MycoShield were introduced into the same cage, and all *H. femoralis* individuals were transferred to new plants. Old plants were removed from the cage, but the soil was not replaced because it contained pupae. The study was conducted over 100 days.

### 2.7 Statistical analyses

All statistical analyses were performed using R software [version 4.4.2; (R Core Team, 2024)]. Logistic regression analysis was performed using the glm function (family = binomial) in R version 4.4.2 to model the relationship between elapsed days and infection probability. Data normality was assessed using the Shapiro–Wilk test. Nonparametric tests were applied if normality was violated (*p* < 0.05). All statistical tests were two-tailed, and significance was set at α = 0.05. Data visualization was performed using ggplot2 in R. Fisher’s exact test was used to evaluate the differences in infection rates among the experimental groups. For *post hoc* analysis, the Dunn–Bonferroni test was used. A logit transformation was performed prior to statistical analysis as the sex ratio data were proportional. The Kruskal–Wallis test was used for group comparisons, followed by the Dunn–Bonferroni test for *post hoc* analysis. The number of larvae was analyzed using the nonparametric Friedman test to assess temporal differences (*p* < 0.01). The Dunn–Bonferroni test was conducted as a *post hoc* analysis when significant differences were detected. A repeated-measures analysis of variance was performed to evaluate temporal changes in the number of *H. femoralis* adults. Mauchly’s sphericity test was applied, and the Greenhouse–Geisser correction was used if the assumption of sphericity was violated (*p* < 0.05).

## 3 Results

### 3.1 Reduction of *Wolbachia* and *Rickettsia* infection rates in adult offspring under standard MycoShield treatment

Following a 24-h exposure of adult insects to MycoShield treatment at the standard concentration (1,000-fold dilution), *Wolbachia* infection rates among adult offspring were 6.67% (2/30 individuals) in *L. trifolii* and 3.33% (1/30 individuals) in *H. femoralis*. For *N. formosa*, the *Rickettsia* infection rate was 0% (0/30 individuals) after 24 h of exposure to a honey droplet containing MycoShield at the same concentration.

### 3.2 MycoShield ingestion mechanisms of *L. trifolii* and *N. formosa*

#### 3.2.1 *Liriomyza trifolii* ingests MycoShield through leaf feeding at the adult and larval stages

**FIGURE 1:**
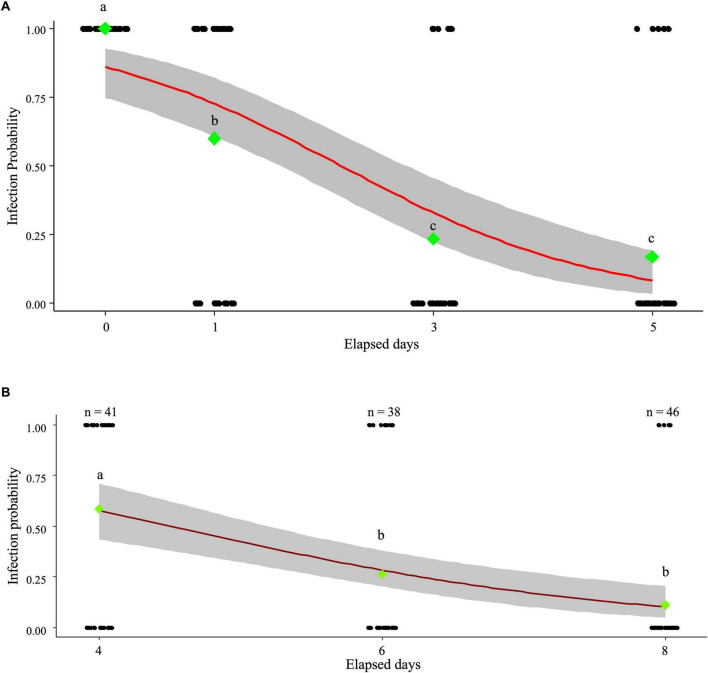
MycoShield uptake by *Liriomyza trifolii* adults and larvae from treated leaves. The data for each individual, with infected individuals represented as one and uninfected individuals as zero, are shown as black-jitter plots. Green diamonds indicate the infection rates for each experimental group. The data were logit-transformed into actual values before statistical analysis, as the infection ratio is proportional to the data. Fisher’s exact test indicated a significant difference (*p* < 0.01), with different letters representing statistical significance (a, b, and c). **(A)** MycoShield uptake experiment results in *L. trifolii* adults. *Liriomyza trifolii* adults were provided with MycoShield-treated leaves for either one or three days. They were then transferred to fresh leaves, and the *Wolbachia* infection rate in the eggs laid on these leaves was assessed. The red line represents the logistic regression curve [$p\,\left(x\right)=\frac{1}{1+e^{-\left(1.8-0.8\,x\right)}}$, *p* < 0.01], whereas the gray area denotes the confidence interval. **(B)** MycoShield uptake experiment results in *L. trifolii* larvae. MycoShields were applied to the leaves on which eggs had been laid, and larvae were collected at four, six-, and eight days post-application to assess infection. The red line represents the logistic regression curve [$p\,\left(x\right)=\frac{1}{1+e^{-\left(3.1-1.1\,x\right)}}$, *p* < 0.01], whereas the gray area denotes the confidence interval.

The next-generation *Wolbachia* infection ratio decreased to 60% after one day of exposure when adult *L. trifolii* were fed by leaves sprayed with MycoShield and allowed to oviposit on fresh leaves. Additionally, the infection rate significantly declined to 23.3% by day three and 16.7% by day five, compared to that before treatment (Figure 1A). The relationship between elapsed days and infection probability was modeled as:


$$p\,\left(x\right)=\frac{1}{1+e^{-\left(1.8-0.8\,x\right)}}$$

where P(x) is the probability of infection at time x. The effect of time was significant (*p* = 5.93 × 10^–9^), indicating a decrease in infection probability over time.

When MycoShield was applied to leaves containing *L. trifolii* larvae, the *Wolbachia* infection rate in *L. trifolii* gradually declined, reaching 10.9% on day eight (Figure 1B). The relationship between elapsed days and infection probability was modeled as:


$$p\,\left(x\right)=\frac{1}{1+e^{-\left(3.1-1.1\,x\right)}}$$

where P(x) is the probability of infection at time x. The effect of time was significant (*p* = 9.24 × 10^–9^), indicating a decrease in infection probability over time.

#### 3.2.2 *Neochrysocharis formosa* does not ingest MycoShield from treated leaves

The *Rickettsia* infection rate in *N. formosa* remained 100% on days one (*n* = 43), three (*n* = 31), five (*n* = 28), and seven (*n* = 45) after exposure to MycoShield-treated leaves, with no males observed. An examination of *N. formosa* pupae revealed a *Rickettsia* infection rate of 100% (*n* = 48) when MycoShield was applied to leaves containing *L. trifolii* parasitized by *N. formosa*. The infection ratio remained unchanged, regardless of the number of days after *N. formosa* was fed dead *L. trifolii* larvae.

#### 3.2.3 *Neochrysocharis formosa* ingests MycoShield from *L. trifolii* larvae through host feeding and parasitism

**FIGURE 2:**
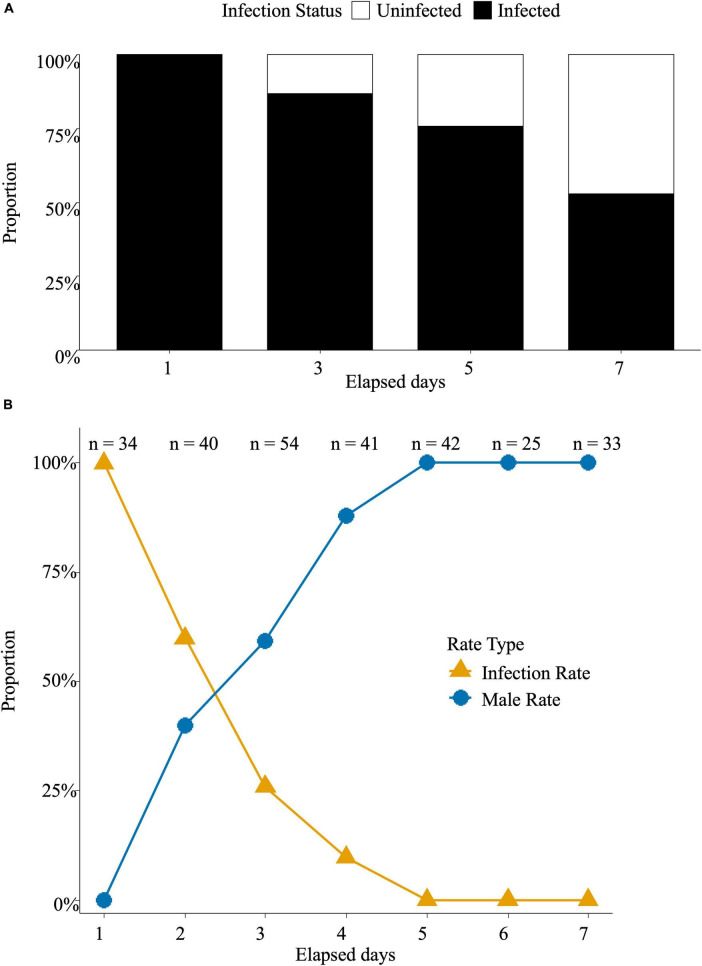
*Neochrysocharis formosa* acquires antibiotics from its host, *L. trifolii* larvae. **(A)** *Neochrysocharis formosa* was introduced and maintained for one, three, five, and seven days after spraying MycoShield on leaves containing *L. trifolii* larvae. They were then transferred to fresh, untreated leaves for oviposition, and whether the collected eggs were infected was determined. Black boxes represent the proportion of infected individuals, whereas white boxes represent the proportion of uninfected individuals. **(B)** *Liriomyza trifolii* adults were allowed to oviposit on MycoShield-treated leaves for five days. When the larvae emerged, *N. formosa* was introduced and allowed to oviposit. *Rickettsia* infection in the next-generation adults was determined. Blue circular plots represent the male sex ratio, whereas orange triangular plots indicate the *Rickettsia* infection rate. The sample size for each time point was denoted as *n* = X.

The *Rickettsia* infection rate in the next generation of *N. formosa* adults on day seven after MycoShield exposure declined to 53% (Figure 2A). *N. formosa* adults acquired MycoShield through host feeding, and the *Rickettsia* infection ratio declined with time. The *Rickettsia* infection rate gradually decreased and reached 0% on day five when *N. formosa* parasitized *L. trifolii* larvae that had ingested MycoShield. The male ratio gradually increased, reaching 100% after five days (Figure 2B).

### 3.3 Effect of MycoShield concentrations

The effects of various MycoShield concentrations on *L. trifolii*, *H. femoralis*, and *N. formosa* are presented in Table 1. At a 100-fold dilution with water, only one *N. formosa* individual was infected, and all tested *L. trifolii* and *H. femoralis* individuals were uninfected. At the standard concentration (diluted 1,000-fold with water), *Wolbachia* was removed from almost all individuals (1.4–2.8% remaining). The infection ratio was increased at low concentrations. The male ratio of *L. trifolii* was not affected by MycoShield, regardless of the concentration. However, for *H. femoralis* and *N. formosa*, the male proportion gradually increased as the concentration decreased.

**TABLE 1:** Symbiont infection rates and female ratios in the next generation of three insect species following MycoShield treatment (*Wolbachia* for *L. trifolii* and *H. femoralis*; *Rickettsia* for *N. formosa*).

Species	Treatment	Infection ratio^1^	Female ratio^2^
*L. trifolii*	*100	0.0%^a^	45.8%
	*1,000	2.8%^ab^	47.2%
	*10,000	54.2%^abc^	43.1%
	*100,000	91.7%^bc^	47.2%
	*1,000,000	95.8%^c^	52.8%
	Water	100.0%^c^	45.8%
*H. femoralis*	*100	0.0%^a^	0.0%^d^
	*1,000	1.4%^a^	1.4%^d^
	*10,000	38.9%^ab^	37.5%^de^
	*100,000	91.7%^ab^	38.9%^de^
	*1,000,000	100.0%^b^	43.1%^de^
	Water	100.0%^b^	100.0%^e^
*N. formosa*	*100	1.4%^a^	1.4%^d^
	*1,000	1.4%^a^	1.4%^d^
	*10,000	51.4%^ab^	43.1%^de^
	*100,000	91.7%^ab^	38.9%^de^
	*1,000,000	97.2%^b^	97.2%^e^
	Water	100.0%^b^	100.0%^e^

^1^The infection ratio for each species was transformed into real values using logit transformation before statistical analysis. Different letters indicate significant differences in application concentrations within the same species (^a,^ ^b, c^Dunn–Bonferroni test, *p* < 0.01).

^2^The sex ratio for each species was transformed into real values using logit transformation before statistical analysis. Different letters indicate significant differences in application concentrations within the same species (^d, e^Dunn–Bonferroni test, *p* < 0.01). The Kruskal–Wallis test yielded a non-significant result for the female ratio (*p* > 0.05) for *L. trifolii*, which lacks letter annotations. Each experimental group contained 12 members, and all experiments were repeated six times. *Values indicate dilution factors of MycoShield (e.g., *100 = 100-fold dilution); higher values correspond to lower concentrations.

### 3.4 Extinction of *H. femoralis*

**FIGURE 3:**
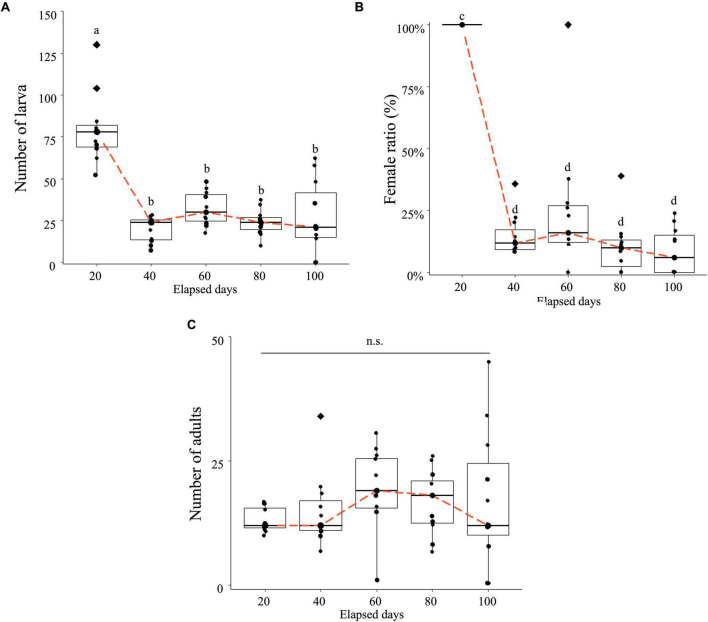
*Hercinothrips femoralis* exhibits a decrease in the female ratio and population size under prolonged MycoShield application. The data obtained from the 11 rearing cages are plotted along with box plots. The orange dashed line connects the median values for each time point, and diamonds represent the outliers. **(A)** Changes in larval count over 100 days of rearing after MycoShield application. *Post hoc* analysis was performed using the Dunn–Bonferroni test, and significantly different groups are indicated by different letters (a and b). **(B)** Changes in the female ratio over 100 days of rearing after MycoShield application. Because the female ratio is proportional to the data, it was logit-transformed into real values before statistical analysis. *Post hoc* analysis was conducted using the Dunn–Bonferroni test and significantly different groups are indicated by different letters (c and d). **(C)** Changes in adult counts after MycoShield application. Repeated measures analysis of variance with this correction revealed no significant differences among the time points (n.s.: *p* = 0.388).

Whether the application of MycoShield affects the population dynamics of *H. femoralis*, in which *Wolbachia* induces parthenogenesis, was investigated. Larval numbers were initially high on day 20 but showed a sharp decline by day 40 and remained low thereafter (Figure 3A). The proportion of adult females was nearly 100% on day 20 but dropped dramatically by day 40, remaining consistently at approximately 20% or lower through day 100 (Figure 3B).

Although adult numbers increased between days 20 and 60, no significant difference was observed across the cages during the entire observation period (Figure 3C). By day 80, three cages exhibited a female ratio of 0%. Among these, two cages (Rep. 5 and Rep. 10) experienced complete extinction by day 100, with both larvae and adults absent (Figure 3C and Table 2). Notably, the cages in which larvae had disappeared were identical to those in which adult populations ultimately became extinct.

**TABLE 2:** Effects of prolonged MycoShield application on female ratio and population size for *Hercinothrips femoralis*.

	20 days			40 days			60 days			80 days			100 days		
Rep	Larva^a^	Adults	Female ratio^c^	Larva^b^	Adults	Female ratio^d^	Larva^b^	Adults	Female ratio^d^	Larva^b^	Adults	Female ratio^d^	Larva^b^	Adults	Female ratio^d^
1	130	10	100%	24	34	11.8%	42	22	22.7%	34	25	12.0%	21	12	16.7%
2	78	11	100%	19	20	10.0%	33	26	0.0%	10	26	15.4%	48	28	0.0%
3	80	12	100%	25	18	22.2%	30	27	25.9%	22	12	8.3%	16	12	0.0%
4	104	11	100%	28	7	14.3%	17	16	37.5%	37	18	11.1%	58	34	20.6%
5	72	17	100%	25	14	35.7%	26	31	16.1%	17	7	0.0%	0	0	0.0%
6	62	15	100%	28	11	9.1%	27	15	6.7%	24	18	38.9%	62	45	13.3%
7	79	12	100%	26	16	12.5%	44	25	28.0%	28	20	10.0%	14	17	5.9%
8	84	16	100%	14	12	8.3%	48	18	11.1%	21	22	4.5%	35	21	23.8%
9	68	12	100%	13	10	20.0%	23	15	13.3%	26	14	14.3%	20	8	12.5%
10	70	12	100%	10	12	8.3%	22	1	100.0%	18	8	0.0%	0	0	0.0%
11	52	16	100%	7	11	9.1%	39	19	15.8%	24	13	0.0%	21	12	0.0%

^a,b^Larval count: The Shapiro–Wilk test indicated non-normality (*p* < 0.01). Therefore, the Friedman test, a nonparametric test for paired data, was conducted (*p* < 0.01). *Post hoc* analysis using the Dunn–Bonferroni test identified significant differences between groups, which are denoted by different letters.

^c,d^Female ratio: As the female ratio is proportional data, it was logit-transformed into real values before statistical analysis. The Friedman test indicated a significant difference (*p* < 0.05), and *post hoc* analysis using the Dunn–Bonferroni test identified significant differences between groups. Adult count: The Shapiro–Wilk test indicated non-normality (*p* > 0.05). Mauchly’s test was performed to assess the sphericity assumption for repeated measures analysis of variance (ANOVA). The result indicated a violation of sphericity (*p* < 0.05). Therefore, a Greenhouse–Geisser correction was applied to adjust the degrees of freedom. The repeated measures ANOVA with the correction revealed no significant differences among time points (*p* = n.s.). To assess the effects of prolonged MycoShield application, 20 newly emerge d female adults (within one day of eclosion) were released, and MycoShield was applied every 20 days for 100 days.

These results indicate that MycoShield treatment drastically reduced the reproductive potential and population size of *H. femoralis* under caged conditions. Complete extinction occurred in two out of eleven cages within 100 days, likely due to genetic drift following severe population bottlenecks.

## 4 Discussion

*Wolbachia* was almost eliminated from *L. trifolii* and *H. femoralis* when MycoShield was sprayed at the standard concentrations on kidney bean leaves. Additionally, *Rickettsia* was completely removed (Table 1) when *N. formosa* was fed MycoShield with honey. Although only one agrochemical antibiotic was tested, two species of endosymbionts were successfully removed. A wide variety of agrochemical antibiotics exist globally, and *Wolbachia* and *Rickettsia* infect 52% and 24% of arthropod species, respectively (Weinert et al., 2015; Zug and Hammerstein, 2012), with numerous other symbionts also present in many species (Douglas, 1998; Kikuchi et al., 2007; Rupawate et al., 2023). These findings suggest that agrochemical antibiotics can be used as effective tools to manage agricultural pests.

The application of MycoShield to leaf surfaces resulted in a significant reduction in *Wolbachia* infection rates of *L. trifolii* and *H. femoralis* adults, suggesting that *Wolbachia* was eliminated through the gradual uptake of the antibiotic by adults (Figure 1A and Table 1). Although *L. trifolii* larvae burrow into the leaves, the application of MycoShield to the leaf surface also reduced the *Wolbachia* infection rate (Figure 1B). This finding suggests that *Wolbachia* were eliminated as a result of larval ingestion of MycoShield, which penetrated the leaf tissue.

The route of MycoShield uptake by *N. formosa*, which parasitizes *L. trifolii*, was also investigated. *N. formosa* did not ingest antibiotics upon contact with its leaves. However, when its mouthparts came into contact with *L. trifolii* exposed to MycoShield or when it fed on the host’s body fluids or internal tissues, antibiotic absorption occurred, leading to a reduced infection rate (Figures 2A, B). These findings suggest that antibiotic uptake by *N. formosa* is indirect, occurring through exposure to an antibiotic-treated host. Key stages of this process include feeding on the host and larval consumption of infected tissues.

In agricultural settings, MycoShield is typically diluted 1,000-fold with water before application. At this concentration, the *Wolbachia* infection rate in *L. trifolii* decreases to 2.8%, the *Wolbachia* infection rate in *H. femoralis* decreases to 1.4%, and the *Rickettsia* infection rate in *N. formosa* decreases to 1.4% (Table 1). These results indicate that MycoShield application in agricultural settings can effectively eliminate symbiotic bacteria from insects.

When *N. formosa* was exposed to MycoShield diluted 1,000-fold, *Rickettsia* was eliminated, resulting in the loss of thelytoky parthenogenesis and the production of male offspring (Figure 2B). This finding suggests that MycoShield may also influence *N. formosa* populations in agricultural settings, particularly in controlling leaf miners. Some parasitoid wasps are commercially available as biological control agents, playing a crucial role in pest population management via parasitism (van Lenteren, 2012). As female wasps are essential for successful biological control, careful selection of pest management methods is necessary to avoid compromising parasitoid populations.

Although selective elimination of *Wolbachia* without affecting *Rickettsia* would be ideal for preserving the biological control capacity of *N. formosa*, currently no antibiotics are known that can achieve such specificity. This is because *Wolbachia* and *Rickettsia* are both members of the Alphaproteobacteria (Dunning Hotopp et al., 2006) and share many fundamental molecular targets, such as ribosomal components and replication machinery (Andersson et al., 1998; Ioannidis et al., 2007; Zug and Hammerstein, 2015). Consequently, the application of broad-spectrum antibiotics like tetracycline inevitably impacts both symbionts (Nguyen et al., 2014). However, previous studies in aphids have demonstrated selective removal of specific endosymbionts through dose-dependent antibiotic treatments—e.g., *Serratia* via ampicillin and *Buchnera* via rifampicin (Koga et al., 2007). These findings suggest that with careful optimization of antibiotic type and concentration, selective targeting of symbionts like *Wolbachia* and *Rickettsia* may be achievable in other insect systems. Further research to explore such possibilities could significantly improve the integration of antibiotic-based approaches with biological control programs.

To evaluate long-term suppression, we conducted a 100-day experiment in which MycoShield was applied every 20 days at a field-relevant concentration (1,000-fold dilution). In theory, eliminating thelytoky parthenogenesis-inducing *Wolbachia* should result in all-male offspring, leading to population collapse (Table 1, female ratio: 1.4%). This outcome was observed in two plots where both larvae and adults were absent by day 100 (Figure 3C). However, in most cages, female production persisted and complete suppression was not achieved, likely due to partial clearance of symbionts. Although Table 1 shows a sharp decline in the female ratio among next-generation adults following treatment, Table 2 indicates that female ratios remained at 10–20% in most cages during the 40–100 day period. This discrepancy can be explained by several biological and methodological factors.

First, the life cycle of *H. femoralis* progresses from egg to adult in approximately 18–20 days at 27°C (Laughlin, 1971). Since insect development generally slows down at lower temperatures, the cycle at our experimental condition (25°C) is expected to be slightly longer. Therefore, the 100% female ratio observed on day 20 likely reflects surviving F_0_ females rather than newly emerged F_1_ individuals. Second, although larvae likely ingest antibiotics during feeding, the efficacy of symbiont elimination depends on both the timing and cumulative dose of exposure (Koga et al., 2007; Shan et al., 2016). Since plants were replaced every 20 days, all larval stages likely encountered treated leaves; however, variability in ingestion among instars may have led to insufficient antibiotic uptake in some individuals. Moreover, as *H. femoralis* pupates on the soil surface, prepupae and pupae may have escaped continued exposure, especially if they entered these protected stages shortly after initial ingestion. This partial exposure scenario may explain why endosymbionts persisted in some individuals.

Interestingly, five cages had only male adults by day 100, suggesting that eradication may occur over a longer timeframe. These male-only populations were effectively reproductively sterile, as no functional females remained to sustain reproduction. This condition reflects a severe reproductive bottleneck, where antibiotic-induced elimination of symbionts disrupts thelytokous parthenogenesis. In such small, male-biased groups, genetic drift further impedes the chance of symbiont persistence or spontaneous recovery, ultimately leading to local extinction. These findings highlight the importance of optimizing treatment timing and frequency to achieve consistent disruption of symbiont-mediated reproduction. However, field conditions may introduce additional variables—such as environmental fluctuation and immigration—that could buffer or counteract this process, warranting further investigation in open settings.

The diverse beneficial roles of symbiotic microorganisms in insect hosts are well-documented. The partial or complete suppression of these microorganisms can result in nutrient deficiencies, weakened immunity, impaired plant defense mechanisms, and increased susceptibility to pathogens, parasites, and predators. Such disruptions may contribute to the partial or complete elimination of pest populations in the field by disrupting essential host functions (Rupawate et al., 2023; Gonella and Alma, 2023). Consequently, targeting symbiotic microorganisms is a promising strategy for integrated pest management.

This study used MycoShield (17.0% oxytetracycline) as the antibiotic treatment; however, numerous antibiotic agrochemicals are commercially available worldwide (McManus et al., 2002). Given the diversity of these compounds, alternative antibiotic agrochemicals may exert broader or more potent antibacterial effects, not only against obligate endosymbionts but also against gut-associated microbiota and mycetocyte-residing microorganisms in infected insects. Additionally, their effects on insect physiology, feeding behavior, and population dynamics warrant further investigation. Investigating the differential effects of various agrochemical antibiotics on insect pests and their associated microbiota is essential for optimizing symbiont-targeted pest control strategies.

## Data Availability

The original contributions presented in this study are included in this article/Supplementary material, further inquiries can be directed to the corresponding authors.
