# Supplementary material for: Antibiotic agrochemical treatment reduces endosymbiont infections and alters population dynamics in leafminers, thrips, and parasitoid wasps
Source: Front Microbiol. 2025 Jun 10;16:1605308. doi: 10.3389/fmicb.2025.1605308 (PMC12185494; doi:10.3389/fmicb.2025.1605308)
Supplement: Supplementary file 1 [file Supplementary_file_1.docx]

Supplementary figure and tables

**Antibiotic agrochemical treatment reduces endosymbiont infections and alters population dynamics in leafminers, thrips, and parasitoid wasps**

**Yuta Ohata^1*^,** **Yohsuke Tagami^2^**

^1^ Gifu University, United Graduate School of Agricultural Science, Gifu, Japan

^2^ Shizuoka University, Graduate School of Agriculture, Shizuoka, Japan

*** Correspondence:**

Yuta Ohata, Yohsuke Tagami

[ohata.yuta@gmail.com](mailto:ohata.yuta@gmail.com); tagamiy@gmail.com

**Keywords: endosymbiont, antibiotic agrochemical, pest control, *Wolbachia*, natural enemy insect**

Running title: Antibiotic biochemicals for endosymbiont-infected insects


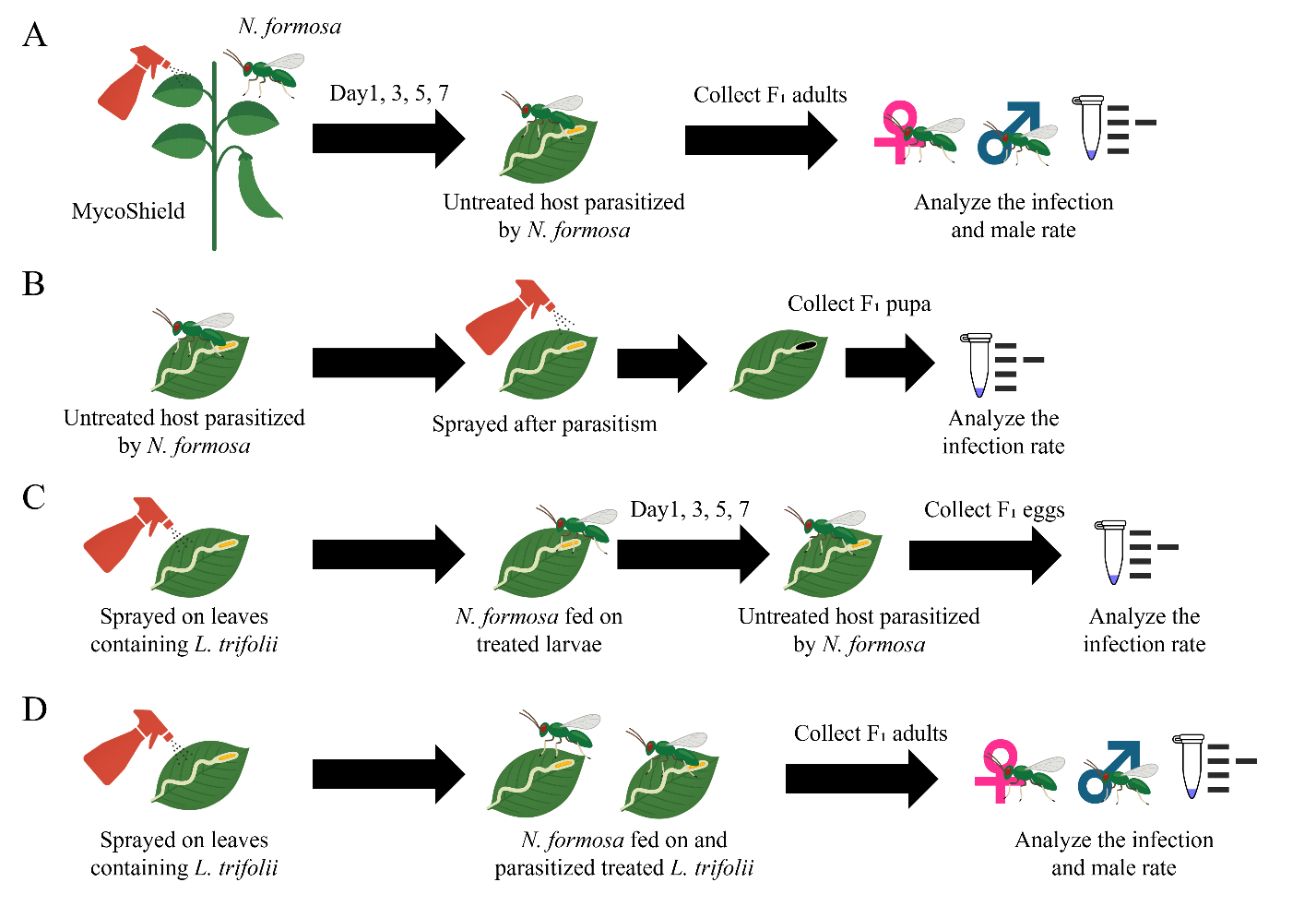
**Supplementary Figure 1.** **Experimental designs for evaluating MycoShield uptake routes and their effects on *Wolbachia* infection and sex ratio in *Neochrysocharis formosa***

**(A)** MycoShield was sprayed onto unparasitized leaves, and *N. formosa* adults were released. Adults were transferred to untreated *Liriomyza trifolii*-parasitized plants on days 1, 3, 5, and 7. F₁ adults were collected to analyze Wolbachia infection and male ratio.
**(B)** MycoShield was applied after parasitism had occurred. F₁ pupae were collected to assess Wolbachia infection rate.
**(C)** *N. formosa* adults fed on *L. trifolii* larvae that had been exposed to MycoShield. On days 1, 3, 5, and 7, the adults were transferred to untreated hosts, and F₁ eggs were collected for infection analysis.
**(D)** A combined route: *N. formosa* adults fed on and parasitized *L. trifolii* larvae reared on MycoShield-treated leaves. F₁ adults were collected to evaluate both infection and male ratio.
